# Echocardiographic Predictors of Ventricular Arrhythmias Post-Automatic Implantable Cardioverter–Defibrillator Implantation

**DOI:** 10.3390/jcdd12120476

**Published:** 2025-12-03

**Authors:** Mehmet Harapoz, Yan Stanislaw Andrzej Zochowski, Siddharth J. Trivedi, Saurabh Kumar, Liza Thomas

**Affiliations:** 1 Department of Cardiology, Westmead Hospital, Parramatta, NSW 2145, Australia; 2 Westmead Clinical School, University of Sydney, Parramatta, NSW 2145, Australia; 3 Westmead Applied Research Centre, University of Sydney, Parramatta, NSW 2145, Australia; 4 South Western Sydney Clinical School, University of New South Wales, Liverpool, NSW 2170, Australia

**Keywords:** ventricular arrhythmia, echocardiography, defibrillator

## Abstract

(1) Background: Ventricular arrhythmias (VAs) are a leading cause of morbidity and mortality in ischemic and non-ischemic heart disease. While automated implantable cardioverter–defibrillators (AICDs) are standard treatment for high-risk patients, predicting future VA post-implantation remains limited. This study evaluated echocardiographic and strain parameters for predicting VA risk in AICD recipients. (2) Methods: This retrospective cohort study included patients who underwent AICD implantation at Westmead Hospital, New South Wales, Australia (January 2014–May 2024). Pre-implant transthoracic echocardiograms (TTEs) were analysed for structural and functional parameters, including left-ventricular (LV) ejection fraction (LVEF), LV global longitudinal strain (GLS), mechanical dispersion (MD), and delta contraction duration (DCD). VA events, defined as appropriate AICD shock or anti-tachycardia pacing, were identified from electronic medical records and device checks. Univariate and multivariate Cox regression analyses were performed. (3) Results: Among 242 patients, 98 experienced VA events. Increased LV end-diastolic diameter, indexed LV mass, and right-ventricular basal diameter were associated with VA events (*p* < 0.05), whilst LVEF and GLS were not. LV dyssynchrony was greater in affected patients (MD 69.2 ms vs. 63 ms, *p* = 0.036; DCD 288.8 ms vs. 246.4 ms, *p* = 0.010). DCD was an independent predictor of VA events (HR 1.003; 95% CI: 1.000–1.006; *p* = 0.022). (4) Conclusions: DCD may improve risk stratification in AICD patients.

## 1. Introduction

Ventricular arrhythmias (VAs) are a leading cause of morbidity and mortality in ischemic and non-ischemic heart disease [1,2]. Patients identified to be at high risk of VA are treated with an automated implantable cardioverter–defibrillator (AICD) [1]. Patient selection for AICD is broadly divided into primary prevention and secondary prevention, based on current guidelines [3,4]. Secondary prevention includes patients with prior sustained, symptomatic ventricular tachycardia (VT) or ventricular fibrillation (VF), while primary prevention refers to individuals with cardiac disease at high risk of VA [3,5]. Current guideline indications for AICD treatment are based on prior life-threatening VA or high-risk features, primarily based on echocardiographic reduced left-ventricular ejection fraction (LVEF) [3,4]. However, patient selection is important as AICDs can cause complications unrelated to VA [6].

Patients with recurrent VA despite an AICD remain at elevated risk of mortality and heart failure despite effective VA termination [7]. There has been growing interest in predictors of VA post-AICD, using tools such as electrocardiograms and machine learning [8,9]. Despite current guidelines for patient selection, which are heavily reliant on LVEF, the utility of other echocardiographic parameters remains relatively unexplored in the prediction of further VA events post-AICD treatment.

This study aims to evaluate echocardiographic parameters, including traditional and speckle tracking-derived strain parameters, in patients undergoing AICD implantation to assess the association with future VA. We hypothesised that greater systolic dysfunction and left-ventricular (LV) dyssynchrony on echocardiography would correlate with higher subsequent VA risk.

## 2. Methods

We retrospectively reviewed all patients undergoing an AICD implantation at Westmead Hospital, New South Wales, Australia (January 2014–May 2024), for both primary and secondary prevention, and consecutively recruited patients who were undergoing an index AICD implantation. Patients were excluded from the study if a pre-implant comprehensive transthoracic echocardiogram (TTE) was not performed at our centre within 12 months prior to device implantation or if they had severe valvular disease or prosthetic valves. Several patients were referred from other centres for AICD insertion, and TTEs were not available for performing measurements. Demographic, clinical, and follow-up data including device checks post-AICD implantation for VA events were collected for all patients from our institution’s electronic medical records with standardised chart review protocols used by the investigators. The primary endpoint of our study was the first VA event for included patients. This study followed the STROBE criteria, with the completed checklist incorporated in the Supplementary Materials [10].

### 2.1. Echocardiography

Comprehensive TTEs were performed for patients using commercially available ultrasound systems (General Electric Vivid E9/E95; Horton, Norway), including 2-dimensional, colour, and Doppler echocardiography, in accordance with the American Society of Echocardiography guidelines [11]. Dedicated apical left-ventricular (LV) views were obtained at high frame rates (>55 frames per second) and stored as raw digital data for offline analysis by two experienced cardiology fellows blinded to patient outcomes.

LV interventricular septal and posterior wall thicknesses were measured using parasternal long-axis images in end-diastole [11]. Biplane LV end-diastolic and end-systolic volumes from apical 4- and 2-chamber views were measured using a modified method of disks, allowing for the calculation of LVEF [11]. LV-indexed mass (LVMI) was obtained using the Devereux formula and then indexed to the body surface area [12].

LV diastolic function was assessed from peak E- and A-wave velocities obtained with pulsed-wave Doppler, with the sample volume placed at the mitral leaflet tips [11]. Septal and lateral e’ velocities were obtained from the septal and lateral mitral annulus, respectively, using tissue Doppler, and calculating an average of the septal and lateral e’ velocities. The E/e’ ratio was obtained as the ratio of peak E velocity and average e’ velocity.

A two-dimensional speckle tracking strain analysis of the LV was performed using specialised offline computer software (EchoPac Version 206; GE systems; GE Healthcare; Milwaukee, WI, USA). Global longitudinal strain (GLS) was evaluated from the 3 standard LV apical views (apical 4-, 2-, and long-axis). The endocardium was traced, and a region of interest was adjusted to fit the myocardial thickness, using offline software, providing an 18-segment LV model (6 segments from each apical view). Manual adjustment was performed for segments failing to track, allowing 2 segments to be excluded from any apical view if appropriate tracking was not achieved. Patients in sinus rhythm had 3 measurements and those in atrial fibrillation had 5 measurements averaged.

**Figure 1:**
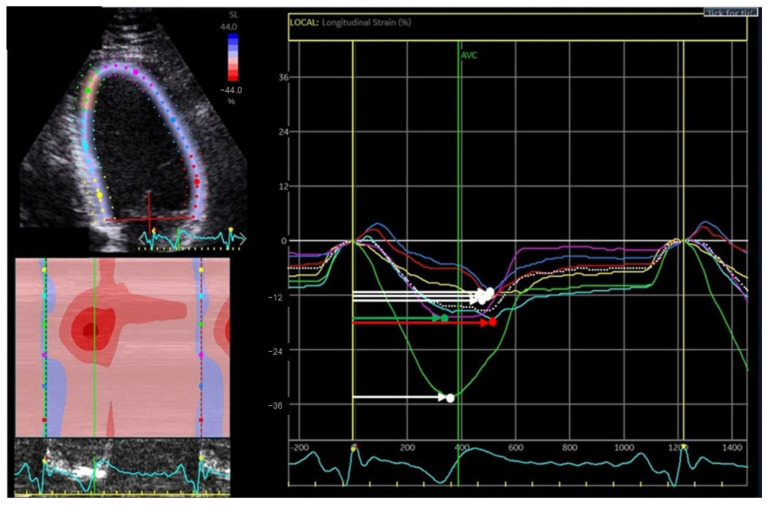
Calculation of left-ventricular mechanical dispersion and delta contraction duration. Mechanical dispersion is calculated as the standard deviation of time from QRS on electrocardiography to the peak negative strain for all segments (arrows). Delta contraction duration is the difference between the time from QRS on electrocardiography to peak negative strain of the longest interval (red arrow) and the shortest interval (green arrow).

The 18-segment LV strain analysis was used for LV mechanical dispersion (MD) calculation, defined as the standard deviation of the time from QRS to peak negative strain from all LV segments (Figure 1) [13]. Myocardial segmental contraction duration was measured on strain analysis as the time from QRS complex onset until maximum negative myocardial shortening of each segment [13]. Dyskinetic (positive strain) and akinetic segments (zero strain) were excluded [13]. LV MD was used to assess LV dyssynchrony for each patient. Additionally, delta contraction duration (DCD), defined as the difference between the longest and shortest time-to-peak contraction from 18 myocardial segments, was measured [13].

### 2.2. AICD Checks

Device checks for all included patients were reviewed from the date of implantation till VA occurrence or the last available device check at our institution (30 May 2024). VAs on device checks were defined as appropriate AICD shock or anti-tachycardia pacing for VT/VF [14]. These had been previously adjudicated by an experienced cardiac technician and the reviewing physician, a senior cardiac electrophysiologist, and were confirmed by an investigator blinded to the TTE measurements.

### 2.3. Statistical Analysis

Data normality was tested with the Shapiro–Wilk test. Mean and standard deviation or median and interquartile range were used to express continuous variables. Numbers and percentages were used to express categorical variables. TTE parameters between groups were compared using independent *t*-tests for continuous variables and Chi-squared tests for categorical variables. TTE parameters associated with VA occurrence were assessed using Cox regression univariate analysis. Furthermore, multivariate Cox regression analysis was performed, incorporating statistically significant parameters from the Cox regression univariate analysis. Log-minus-log plots were used to ensure no violation of the Cox proportional hazards assumption model. Receiver operating characteristic (ROC) curve analysis was performed. The study endpoint was VA occurrence, assessed on patient device checks. Missing TTE parameters were excluded pairwise in the analysis. A *p*-value < 0.05 was considered statistically significant. Statistical analysis was performed using the SPSS software version 29.0 (SPSS, Inc., Chicago, IL, USA).

## 3. Patient and Public Involvement

The patients and the public were not involved in the planning or conduct of this study.

## 4. Results

**Figure 2:**
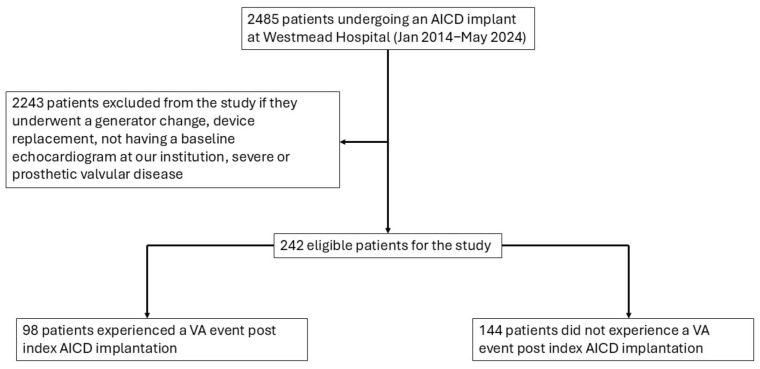
Patient identification and selection flowchart. Abbreviations: AICD = automated implantable cardioverter–defibrillators and VA = ventricular arrhythmia.

A total of 242 patients who underwent AICD implant between January 2014 and May 2024, with a comprehensive TTE at our centre and meeting the inclusion criteria, were recruited to this retrospective study, from a total of 2485 patients who were screened. Of these, 117 patients had ischemic cardiomyopathy (ICM), while 125 patients had non-ischemic cardiomyopathy (NICM). There were 171 patients who underwent an AICD implant for primary prevention and 71 patients for secondary prevention. In total, 209 patients within this cohort were on at least one anti-arrhythmic drug. The cohort was mainly male (75.6%), with a mean age of 61 ± 16 years. The median time from TTE to AICD implant was 48 days (interquartile range 336 days). The mean follow-up time/time of VA event in the cohort was 17.6 ± 17.7 months and our median follow-up time was 11.1 months (interquartile range 26 months). A total of 98 patients were identified with VA events on their device checks. The demographic and TTE parameters of the entire cohort are presented in Table 1 and the patient identification and selection flowchart is presented in Figure 2.

All pre-implant AICD TTEs were re-analysed, and offline measurements were performed by two investigators who were blinded to the VA occurrence data. Some parameters were not measured due to missing images or poor image quality (septal and lateral e’ (*n* = 12), peak E-wave velocity (*n* = 5), right-ventricular (RV) s’ velocity (*n* = 13), TAPSE (*n* = 10), and RV basal diameter (*n* = 3). LV strain parameters were available for all patients. There was only one patient in atrial fibrillation at the time of the echocardiogram.

We examined the association between echocardiographic parameters and VA episodes post-AICD implant (Table 2). A total of 98 patients experienced a VA event after AICD implantation and 144 did not. There were no significant differences in age, sex, cardiomyopathy type, AICD indication, anti-arrhythmic therapy use, or anthropometric measurements between the groups (Table 2).

Of the traditional echocardiographic parameters evaluated, LV end-diastolic diameter (LVEDD) and LVMI were increased in patients who experienced VA events post-AICD implant compared to those who did not (Table 2). However, LV end-diastolic volume indexed (LVEDVI) was increased in both groups without a statistically significant difference.

LV end-systolic diameter and volume, interventricular septum, and posterior wall thickness demonstrated trends without statistically significant differences between the two groups (Table 2). Not surprisingly, LVEF was reduced in both groups, but did not demonstrate any statistically significant difference (Table 2). Similarly, LV strain analysis demonstrated that LV GLS, although reduced in both groups, was not significantly different. Measures of LV dyssynchrony, including MD and DCD, demonstrated a statistically significant difference (Table 2) with both MD (69.2 vs. 63 ms, *p*-value 0.036) and DCD (288.8 vs. 246.4 ms, *p*-value 0.010) increased in AICD patients who experienced VA events compared to AICD patients without VA events.

Diastolic function parameters were analysed in both groups of patients. Mitral inflow parameters including peak E-wave velocity, peak A-wave velocity, and E/A ratio were not statistically significant between AICD patients who experienced VAs and those that did not (Table 2). Similarly, tissue Doppler septal, lateral, and average e’ velocities and E/e’ ratio did not demonstrate a statistically significant difference between the two groups (Table 2).

Left-atrial (LA) and RV echocardiographic parameters were also reviewed. LA-indexed volume was increased in both groups, without a statistically significant difference (Table 2). RV basal diameter was increased in patients with an AICD who experienced VAs compared to AICD patients who did not experience VAs (42 vs. 39 mm, *p*-value 0.007) (Table 2). However, RV functional parameters including RV s’ velocity and tricuspid annular plane systolic excursion (TAPSE) demonstrated normal RV systolic function, with no significant differences between the two AICD groups (Table 2).

Cox regression univariate and multivariate analyses were performed on a per-unit-change basis (Table 3). Univariate analysis demonstrated that LVEDD (*p* = 0.015; hazard ratio (HR) 1.290, 95% CI 1.051–1.583), LVMI (*p* < 0.001; HR 1.004, 95% CI 1.003–1.006), RV basal diameter (*p* = 0.008; HR 1.243, 95% CI 1.058–1.460), MD (*p*-value = 0.009; HR 1.012, 95% CI 1.003–1.020), and DCD (*p*-value = 0.002; HR 1.002, 95% CI 1.001–1.004) were univariate determinants of VA. However, multivariate Cox regression analysis of univariate parameters demonstrated that only LVMI (*p*-value < 0.001; HR 1.005, 95% CI 1.003–1.006) and DCD (*p*-value = 0.022; HR 1.003, 95% CI 1.000–1.006) demonstrated an independent association with VA occurrence.

**Figure 3:**
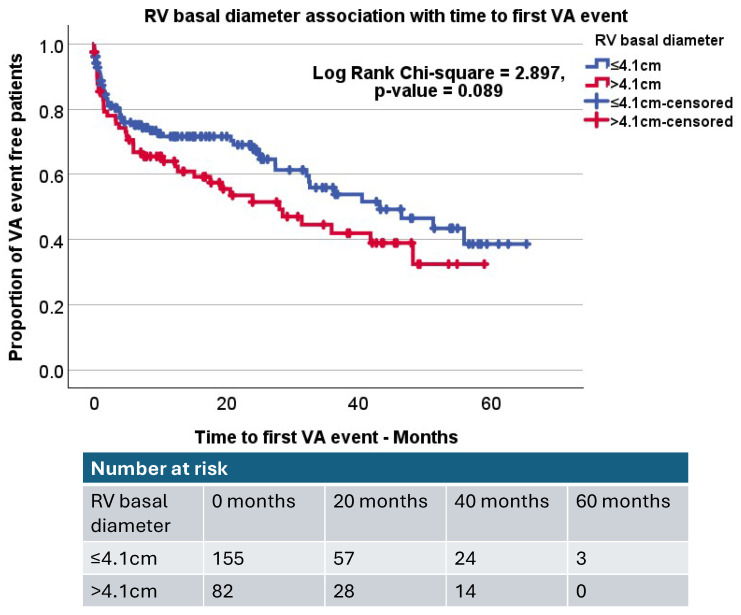
Kaplan–Meier curve demonstrating the impact of normal RV basal diameter (RV basal diameter ≤ 4.1 cm) compared to a dilated RV (RV basal diameter > 4.1 cm). Log rank *p*-value = 0.089.

A Kaplan–Meir analysis was performed for the parameters identified as significant on univariate analysis. LVMI was assessed within the AICD patients using standard gender-specific cut-off values of 115 g/m^2^ for males and 95 g/m^2^ for females. Interestingly, only males with an increased LVMI were more likely to experience VA events compared to males without (Log rank, *p* < 0.001), but females did not demonstrate this association (Log rank, *p* = 0.152). Further Kaplan–Meier analysis demonstrated that AICD patients with a dilated RV (RV basal diameter > 4.1 cm) had a non-significant trend towards experiencing VA events (Log rank, *p*-value = 0.089, Figure 3).

**Figure 4:**
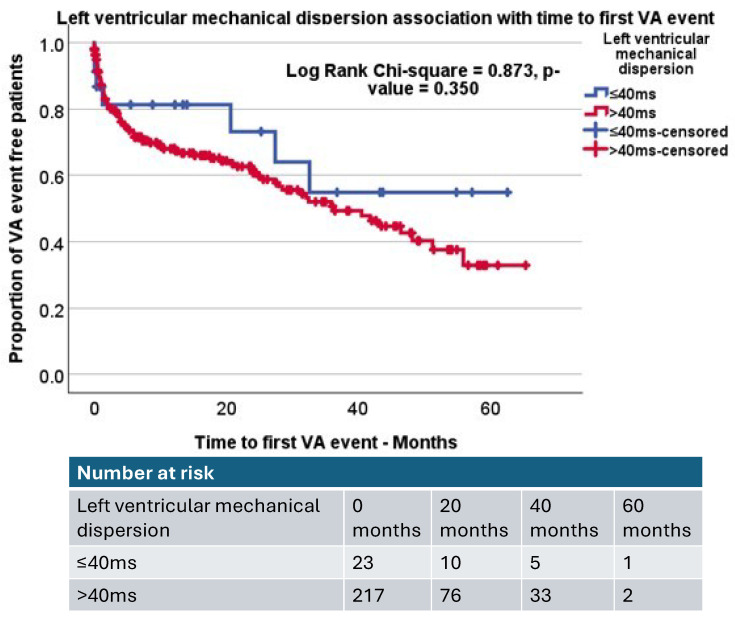
Kaplan–Meier curve demonstrating the impact of increased mechanical dispersion (mechanical dispersion > 40 ms) compared to those with mechanical dispersion within normal limits (mechanical dispersion ≤ 40 ms). Log rank *p*-value = 0.350.

**Figure 5:**
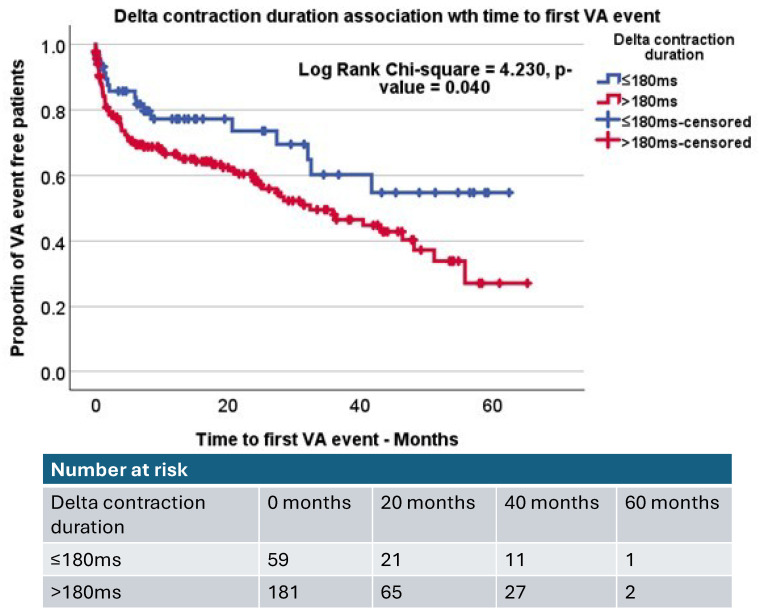
Kaplan–Meier curve demonstrating the impact of increased delta contraction duration (delta contraction duration > 180 ms) compared to AICD patients with reduced delta contraction duration (delta contraction duration ≤ 180 ms). Log rank *p*-value = 0.040.

MD was further assessed with a Kaplan–Meier analysis using a cut-off of 40 ms, the cut-off for MD determined by previous studies, as a possible clinically relevant reference range compared to healthy controls [15,16]. Kaplan–Meier analysis of MD did not demonstrate significant differences between AICD patients with increased mechanical dispersion (MD > 40 ms) compared to those patients without (Log rank, *p*-value = 0.350, Figure 4). We additionally used a cut-off of MD > 70 msec based on a previous study in patients with AICD implantation [15] and again found no significant differences (*p* = 0.983). Kaplan–Meier analysis of DCD demonstrated that AICD patients with increased DCD (DCD > 180 ms, based on prior studies) [15,17] were more likely to experience VA events compared to those with lower DCD times (Log rank, *p*-value = 0.040, Figure 5). ROC analysis demonstrated DCD to be a limited classifier, and sensitivity analysis demonstrated that 180 ms provided a fair sensitivity (sensitivity 83.7% and specificity 70.8%), which was used for this analysis.

## 5. Discussion

Our primary findings demonstrate that structural changes in the LV and RV (with RV dilatation and increased LV mass) and LV dyssynchrony were determinants of future VA events in patients with AICD. LV systolic function including LVEF and GLS were reduced, but similar between groups, and were not determinants of VA events. However, specific strain-derived measures of LV dyssynchrony, MD, and DCD were increased in AICD patients who experienced VA events. VAs pose a significant mortality and morbidity risk in heart disease of varying aetiologies, with AICD therapy providing a significant advance in patient survival of VA events [7].

LVEF and LV GLS were reduced in both groups, which reflects the selected patient cohort, as current guidelines use a significantly reduced LVEF for AICD therapy [3]. However, similarly to other reports, LV systolic function could not identify individuals likely to develop VA events [18]. Historically, LV function, specifically LVEF, has been the main determinant for AICD implantation for VA [3]. While AICDs are lifesaving, they are not benign and can result in physical and psychological consequences for patients [6,19]. Often, once implanted for primary prevention, the individual does not experience a VA event. Although there have been many studies which assess the occurrence of VA events in patients post-AICD implantation, there remains a lack of evidence on the incremental utility of additional echocardiographic parameters which can be used for the risk stratification of patients likely to have VA reoccurrence [7,20,21]. Only structural changes post-AICD implantation with LV and RV dilatation demonstrated an association with future VA events [22,23]. We similarly observed that increased LV mass and LV and RV dilatation demonstrated an association with further VA events, which has been previously demonstrated [22,23,24]. Interestingly, LVMI was demonstrated to have a statistically significant association with VA events despite LV end-diastolic volume (EDV) and septal and posterior wall thickness not reaching statistical significance between AICD patients who experienced VA events and those who did not. This could be explained by the Devereux formula that was used for LV mass, with group differences likely driven by the difference in LVEDD that was noted between groups.

Strain-based echocardiographic parameters have allowed further advancements in the assessment of various cardiomyopathies, providing diagnostic and prognostic utility with LV GLS and measures of LV mechanical dyssynchrony, with clinical applications including the prediction of VAs [25]. GLS is a subclinical marker of LV function but did not demonstrate differences between groups. However, strain parameters, which are surrogate markers of LV dyssynchrony, have demonstrated utility in identifying individuals at risk of VA events; indeed, our group has previously demonstrated that strain parameters predict scarring on invasive electroanatomic mapping during VA ablation [26,27]. However, these parameters have not been incorporated into routine risk assessments in patients with AICD implants. Several studies previously evaluated the prognostic utility of MD as a marker of ventricular arrhythmogenicity [14,15,28]. There have been a few studies evaluating the role of MD in the prediction of VA in patients post-myocardial infarction [15,29]. These demonstrated that increased MD was independently associated with VA events in patients with an ischemic cardiomyopathy [15,29]. A study by Haugaa et al. [29] demonstrated the utility of LV GLS as well as LV MD used to demonstrate LV contraction heterogeneity, with LV MD being an independent predictor of VAs. A subsequent study assessed the prognostic value of LV MD in risk stratification for sudden cardiac death and VA, which demonstrated that LV MD ≥ 75 ms was predictive of VA, particularly in patients with moderately reduced LVEF [28]. Furthermore, a comprehensive meta-analysis, including the results from 12 published studies, evaluated the association of LV MD and VA events [14]. This demonstrated that LV MD was increased in patients who had VA events, and LV MD was an independent predictor of VA events [14]. Another study evaluated the prognostic value of LV MD in risk stratification for sudden cardiac death or VA events in patients with LV systolic dysfunction, including both ICM and NICM patients [28]. This demonstrated that LV MD offered further value, with increased LV MD being predictive of sudden cardiac death and VA events while LVEF alone was inferior for this prediction [28]. Even though strain-based echocardiographic parameters including LV GLS and LV mechanical dyssynchrony parameters can be influenced by loading conditions and arrhythmia burden [25], these findings support the notion that mechanical contraction heterogeneity is a surrogate marker that may play a central role in arrhythmogenesis.

Our study confirms the association between parameters of LV dyssynchrony and VA events in patients with AICDs. In this study, we evaluated both ICM and NICM patients who had AICD for primary or secondary prevention. Both MD and DCD were univariate predictors of VA events. However, in contrast to prior studies, MD did not remain an independent predictor in multivariate analysis in our cohort. Instead, DCD emerged as a more robust marker. This distinction may reflect differences in the study population and the timing of imaging. Unlike previous studies, focusing primarily on post-myocardial infarction or general heart failure populations, our study focused on patients undergoing AICD implantation. As such, the ability of DCD to discriminate VA risk more accurately than MD suggests that it may capture more pronounced mechanical heterogeneity. Both prolonged MD and DCD are likely surrogates of the underlying electrical discordance or heterogeneity. DCD represents the temporal difference between the earliest and latest peak segmental contraction, potentially reflecting the most extreme degrees of LV contractile heterogeneity. While MD captures standard deviation across all segments, a more generalised and conservative estimate of intersegmental dyssynchrony, DCD directly identifies the outlier segments, such as the range between the maximum and minimum time extremes, that may contribute disproportionately to arrhythmic substrate and better reflect the extent of LV dyssynchrony than MD.

To our knowledge, this is one of the first studies evaluating DCD in a large cohort of AICD patients, and our findings suggest that DCD may provide additional prognostic information versus MD. Our group has previously reported on alterations in DCD in patients with idiopathic VAs, demonstrating worsening DCD in patients who underwent unsuccessful radiofrequency ablation for ventricular arrhythmias, suggesting that DCD is a marker of substrate abnormality [30]. Furthermore, our group demonstrated DCD to be an independent predictor of VA-recurrence-free survival in ICM and NICM patients undergoing catheter ablation for ventricular tachycardia storm [17]. These findings support a growing body of literature challenging the adequacy of LVEF as the sole parameter for arrhythmia risk stratification [31,32]. Our data suggests that mechanical dyssynchrony metrics may enhance patient selection, improve risk stratification, influence treatment decisions (i.e., the addition of anti-arrhythmic drugs), and determine prognosis. It could be considered an addition to existing risk prediction algorithms, including machine learning, and potentially provide additional precision for improved personalised risk assessment for patients.

Our study did have some limitations. It was a retrospective study with data accuracy limited to what was available in the patients’ medical records from a single centre, which introduced referral bias into our data. A significant number of patients who underwent AICD implantation at our site were external referrals and TTEs were not available. Secondly, we acknowledge that some patients may not have had all their follow-up at our institution and, thus, as later VA events would not be available to us, there was no prospective follow-up within our study design. However, this would contribute to a minority (<5%) of individuals. Thirdly, our study included all patients undergoing index AICD implantation, including cardiac resynchronisation therapy defibrillators, and thus the group is heterogenous, consisting of ICM and NICM patients. Given the limited number of patients in each group, we could not perform subgroup analysis for possible differences in the echocardiographic parameters as predictors of VA events in each group. Additionally, given the retrospective and single-centre design of our study, no external validation was performed.

Cox regression analysis can be susceptible to the risk of overfitting, which can impact the generalisability of our results. We adjusted for five parameters in our multivariable analysis to ensure model stability, proving an event-per-variable ratio of 19.6, suggesting adequate model stability. Finally, our study included a mix of primary and secondary prevention patients, which, again, due to limited numbers, precluded subgroup analysis.

Future large-scale prospective multi-centre studies, focusing on patient recruitment based on cardiomyopathy type, would be required to identify differences between the two groups. Although we have demonstrated statistically significant differences in echocardiographic parameters within this study, we acknowledge that the absolute values did not demonstrate clinically significant values to identify clear cut-offs. This may have been limited by the heterogeneity of the study cohort. Further large-scale trials evaluating patients according to cardiomyopathy type and primary or secondary prevention could provide further insight into this.

## 6. Conclusions

We have demonstrated that echocardiographic parameters associated with VA events in patients with AICD implants include structural (LVEDD, LVMI, and RV basal diameter) and functional parameters, identifying mechanical dyssynchrony (LV MD and DCD). Conventional measures such as LVEF and GLS did not predict future VA. DCD emerged as a robust independent echocardiographic predictor of VA events. These findings highlight the value of incorporating measures of mechanical dyssynchrony, particularly DCD, into routine echocardiographic assessment for improved arrhythmic risk stratification. Future prospective studies are warranted to validate these parameters and explore their role in guiding individualised post-AICD care.

## Figures and Tables

**Table 1:** Demographic, clinical, and echocardiographic parameters of AICD patients.

	All AICD Patients
Patients, n	242
Age, years (SD)	61 [±16]
Male, n (%)	183 (75.6)
NICM, n (%)	125 (51.7)
ICM, n (%)	117 (48.3)
Primary prevention (%)	171 (70.7)
Secondary prevention (%)	71 (29.3)
Anti-arrhythmic therapy use (%)	209 (86.4)
VA, n (%)	98 (40.5)
Time to VA/last follow-up, months	17.6 [17.7]
Atrial fibrillation at time of echocardiogram (%)	1 (0.4)
Height, cm	170.6 [9.5]
Weight, kg	85.3 [22.5]
BMI, kg/m^2^	29.1 [6.9]
BSA, m^2^	1.98 [0.26]
LVEDD, (mm)	57 [9.6]
LVSDD, (mm)	45 [12]
IVS, (mm)	10 [3.1]
PW, (mm)	10 [2.4]
LVMI, (g/m^2^)	164.6 [99.2]
EDV, (mL)	153.5 [65.9]
EDV/BSA, (mL/m^2^)	78.6 [33.9]
Peak E-wave, (cm/s)	79 [26.7]
Peak A-wave, (cm/s)	64.3 [28.3]
E/A ratio	1.48 [0.98]
Septal e’, (cm/s)	5.2 [2.5]
Lateral e’, (cm/s)	7.1 [3.4]
Average e’, (cm/s)	6.2 [3.2]
E/e’	15.6 [8.6]
RV basal diameter, (mm)	40 [10.5]
RV s’ velocity, (m/s)	9.7 [2.9]
TAPSE, (mm)	19 [5.4]
LA volume (indexed), (mL/m^2^)	44.1 [20.3]
LVEF, (%)	41.6 [14.6]
Average GLS, (%)	−11.3 [4.5]
Mechanical dispersion, (ms)	65.5 [22.5]
Delta contraction duration, (ms)	263.6 [125.3]

Values are expressed as either number (percentage) or mean [standard deviation]. Abbreviations: AICD = automated implantable cardioverter–defibrillators; BMI = body mass index; BSA = body surface area; EDV = end-diastolic volume; EDV/BSA = end-diastolic volume indexed; GLS = global longitudinal strain; ICM = ischemic cardiomyopathy; IVS = interventricular septum thickness; LA = left atrium; LVEDD = left-ventricular end-diastolic diameter; LVEF = left-ventricular ejection fraction; LVMI = left-ventricular mass indexed; LVSDD = left-ventricular end-systolic diameter; NICM = non-ischemic cardiomyopathy; PW = posterior wall thickness; RV = right ventricle; SD = standard deviation; TAPSE = tricuspid annular plane systolic excursion; VA = ventricular arrhythmia.

**Table 2:** Differences in demographic, clinical, and echocardiographic parameters in AICD patients with and without ventricular arrhythmia events.

	AICD Patients with VA Post (*n* = 98)	AICD Patients Without VA (*n* = 144)	*p*-Value
Age, years (SD)	61 [±14]	61 [± 17]	0.973
Male, n (%)	78 (79.6)	105 (72.9)	0.235
NICM, n (%)	54 (55.1)	71 (49.3)	0.376
ICM, n (%)	44 (44.9)	73 (50.7)	0.376
Primary prevention (%)	64 (65.3)	107 (74.3)	0.132
Secondary prevention (%)	34 (34.7)	37 (25.7)	0.132
Anti-arrhythmic therapy use (%)	89 (90.8)	120 (83.3)	0.097
Time to VA/last follow-up, months	10.7 [14.3]	22.3 [18.3]	<0.001 *
Height, cm	171.6 [10.3]	169.9 [8.8]	0.175
Weight, kg	88.8 [26.4]	82.9 [19.1]	0.046 *
BMI, kg/m^2^	29.7 [7.5]	28.8 [6.4]	0.307
BSA, m^2^	2 [0.3]	1.96 [0.2]	0.219
LVEDD, (mm)	59 [9]	55 [10]	0.008 *
LVSDD, (mm)	46 [12]	43 [12]	0.061
IVS, (mm)	11 [3]	10 [3]	0.059
PW, (mm)	10 [2]	10 [3]	0.092
LVMI, (g/m^2^)	193.7 [116.5]	144.9 [80.1]	<0.001 *
EDV, (mL)	157.9 [69.8]	150.5 [63.2]	0.395
EDV/BSA, (mL/m^2^)	79.5 [34.4]	77.9 [33.6]	0.718
Peak E-wave, (cm/s)	79.5 [29.9]	78.6 [24.4]	0.783
Peak A-wave, (cm/s)	62.1 [29.6]	65.8 [27.4]	0.368
E/A ratio	1.58 [1.09]	1.42 [0.90]	0.251
Septal e’, (cm/s)	5.1 [2.4]	5.2 [2.6]	0.639
Lateral e’, (cm/s)	6.7 [3.2]	7.3 [3.5]	0.167
Average e’, (cm/s)	5.9 [2.7]	6.5 [3.5]	0.165
E/e’	16.1 [9.2]	15.2 [8.2]	0.425
RV basal diameter, (mm)	42 [14]	39 [7]	0.007 *
RV s’ velocity, (m/s)	9.5 [2.7]	9.9 [3.1]	0.332
TAPSE, (mm)	19 [5.2]	19 [5.5]	0.543
LA volume (indexed), (mL/m^2^)	46.7 [23.1]	42.4 [18]	0.115
LVEF, (%)	42.7 [13.6]	40.9 [15.2]	0.323
Average GLS, (%)	−11 [4.3]	−11.5 [4.6]	0.354
Mechanical dispersion, (ms)	69.2 [25.7]	63 [19.7]	0.036 *
Delta contraction duration, (ms)	288.8 [145]	246.4 [107.2]	0.010 *

Values are expressed as either number (percentage) or mean [standard deviation]. Parameters which were statistically significant between the two groups were highlighted with *. Abbreviations: AICD = automated implantable cardioverter–defibrillators; BMI = body mass index; BSA = body surface area; EDV = end-diastolic volume; EDV/BSA = end-diastolic volume indexed; GLS = global longitudinal strain; ICM = ischemic cardiomyopathy; IVS = interventricular septum thickness; LA = left atrium; LVEDD = left-ventricular end-diastolic diameter; LVEF = left-ventricular ejection fraction; LVMI = left-ventricular mass indexed; LVSDD = left-ventricular end-systolic diameter; NICM = non-ischemic cardiomyopathy; PW = posterior wall thickness; RV = right ventricle; SD = standard deviation; TAPSE = tricuspid annular plane systolic excursion; VA = ventricular arrhythmia.

**Table 3:** Univariate and multivariate Cox regression analyses to identify independent predictors of ventricular arrhythmias in AICD patients.

Variables	Univariate Analysis		Multivariate Analysis	
	HR (95% CI)	*p*-Value	HR (95% CI)	*p*-Value
**LVEDD**	**1.290 (1.051–1.583)**	**0.015**	**0.977 (0.768–1.242)**	**0.848**
LVSDD	1.173 (0.994–1.385)	0.059	N/A	N/A
IVS	1.379 (0.812–2.342)	0.235	N/A	N/A
PW	1.685 (0.810–3.507)	0.163	N/A	N/A
**LVMI**	**1.004 (1.003–1.006)**	**<0.001**	**1.005 (1.003–1.006)**	**<0.001**
EDV	1.002 (0.999–1.005)	0.148	N/A	N/A
EDV/BSA	1.004 (0.998–1.010)	0.181	N/A	N/A
Peak E-wave	1.002 (0.994–1.009)	0.646	N/A	N/A
Peak A-wave	1.001 (0.993–1.009)	0.789	N/A	N/A
E/A ratio	1.061 (0.866–1.301)	0.566	N/A	N/A
Septal e’	0.974 (0.895–1.059)	0.536	N/A	N/A
Lateral e’	0.951 (0.891–1.014)	0.127	N/A	N/A
Average e’	0.946 (0.876–1.020)	0.150	N/A	N/A
E/e’ ratio	1.017 (0.993–1.042)	0.177	N/A	N/A
**RV basal diameter**	**1.243 (1.058–1.460)**	**0.008**	**1.097 (0.938–1.283)**	**0.245**
RV s’ velocity	0.946 (0.882–1.014)	0.117	N/A	N/A
TAPSE	0.986 (0.948–1.025)	0.478	N/A	N/A
LA volume (indexed)	1.004 (0.995–1.014)	0.406	N/A	N/A
LVEF	1.002 (0.989–1.016)	0.741	N/A	N/A
Average GLS	1.029 (0.982–1.078)	0.235	N/A	N/A
**Mechanical dispersion**	**1.012 (1.003–1.020)**	**0.009**	**0.996 (0.980–1.013)**	**0.664**
**Delta contraction duration**	**1.002 (1.001–1.004)**	**0.002**	**1.003 (1.000–1.006)**	**0.022**

Abbreviations: AICD = automated implantable cardioverter–defibrillators; EDV = end-diastolic volume; EDV/BSA = end-diastolic volume indexed; GLS = global longitudinal strain; HR = hazard ratio; IVS = interventricular septum thickness; LA = left atrium; LVEF = left-ventricular ejection fraction; LVEDD = left-ventricular end-diastolic diameter; LVMI = left-ventricular mass indexed; LVSDD = left-ventricular end-systolic diameter; N/A = not available; PW = posterior wall thickness; RV = right ventricle; TAPSE = tricuspid annular plane systolic excursion.

## Data Availability

The data presented in this study are available on request from the corresponding author due to privacy, legal, and ethical reasons.

## Supplementary Materials

The following supporting information can be downloaded at https://www.mdpi.com/article/10.3390/jcdd12120476/s1. STROBE Statement—checklist of items that should be included in reports of observational studies.
